# Immunobiography and the Heterogeneity of Immune Responses in the Elderly: A Focus on Inflammaging and Trained Immunity

**DOI:** 10.3389/fimmu.2017.00982

**Published:** 2017-08-15

**Authors:** Claudio Franceschi, Stefano Salvioli, Paolo Garagnani, Magda de Eguileor, Daniela Monti, Miriam Capri

**Affiliations:** ^1^Institute of Neurological Sciences of Bologna IRCCS, Bologna, Italy; ^2^Department of Experimental, Diagnostic and Specialty Medicine (DIMES), University of Bologna, Bologna, Italy; ^3^Interdepartmental Centre ‘L. Galvani’ (CIG), University of Bologna, Bologna, Italy; ^4^Department of Biotechnology and Life Science, University of Insubria, Varese, Italy; ^5^Department of Experimental and Clinical Biomedical Sciences “Mario Serio”, University of Florence, Florence, Italy

**Keywords:** inflammaging, trained immunity, human aging, macrophages, NK cells, immunobiography

## Abstract

Owing to its memory and plasticity, the immune system (IS) is capable of recording all the immunological experiences and stimuli it was exposed to. The combination of type, dose, intensity, and temporal sequence of antigenic stimuli that each individual is exposed to has been named “immunobiography.” This immunological history induces a lifelong continuous adaptation of the IS, which is responsible for the capability to mount strong, weak or no response to specific antigens, thus determining the large heterogeneity of immunological responses. In the last years, it is becoming clear that memory is not solely a feature of adaptive immunity, as it has been observed that also innate immune cells are provided with a sort of memory, dubbed “trained immunity.” In this review, we discuss the main characteristics of trained immunity as a possible contributor to inflammaging within the perspective of immunobiography, with particular attention to the phenotypic changes of the cell populations known to be involved in trained immunity. In conclusion, immunobiography emerges as a pervasive and comprehensive concept that could help in understanding and interpret the individual heterogeneity of immune responses (to infections and vaccinations) that becomes particularly evident at old age and could affect immunosenescence and inflammaging.

## Introduction: The Immune System (IS) as a Complex System

**Figure 1:**
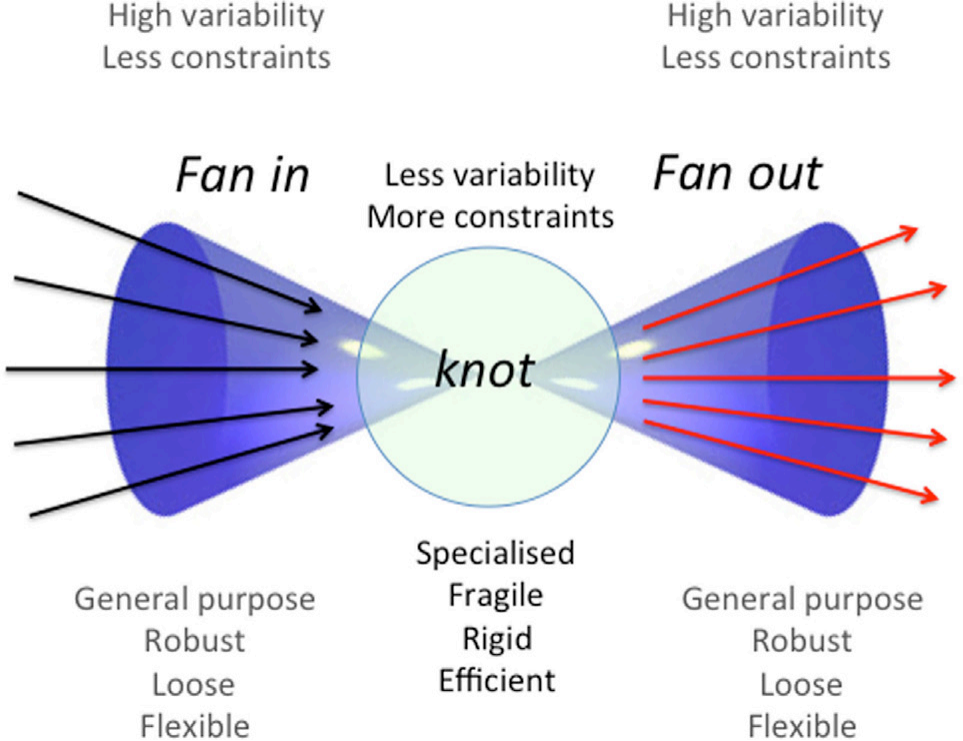
General scheme of a bow-tie architectural module. This operational module is schematized as a bow tie due to the fact that it is composed by a conserved and relatively rigid core (“knot”) of elements and by two wings of inputs and outputs (fan in and fan out, respectively). The core can accept a wide range of inputs that are integrated according to rules and protocols specific for every bow-tie, yielding a wide variety of outputs. These features confer flexibility, robustness, and evolvability to the system. Adapted from Ref. (7).

Life is a continuous exposure to a large variety of threatening and potentially damaging agents collectively indicated as stressors, which can be divided into two basic categories: external and internal stressors. The first category includes not only all sorts of bacteria, viruses, fungi, and parasites but also nutrients that are basically foreign material that are ingested as a source of energy. The second category includes all types of material produced by living organisms as a consequence of cell turnover and metabolism, i.e., cell components or debris, metabolites, and molecular aggregates resulting from incomplete degradation or non-enzymatic reactions, considered as “molecular garbage” (1). All along the evolution, animals from invertebrates to vertebrates have developed adaptive strategies to recognize and neutralize such complex and dynamic combination of stressors that all together represent the “ecospace” where each animal lives (2). On the basis of studies on the evolution of stress response, from invertebrates to mammals (3), we argued that an integrated set of immune–neuro-endocrine responses co-evolved to cope with internal and external stressors (4, 5). It is important to note that, according to this conceptualization, “antigens” can be considered as a particular type of stressors (6). The IS is composed of cells and receptors devoted to the recognition of, and response to antigenic stressors, and is considered a paradigmatic example of complex system. As such, it is characterized by specific features, such as *degeneracy* (the capability of a single receptor to recognize a variety of molecular patterns); *networking* (the capability of IS cells to interact and cross-talk with each other); *plasticity* (the capability to adapt to different situations); and finally, the so-called *bow tie* architecture has been conceptualized to integrate all these characteristics of the IS. This latter is an organizational module that foresees a core of elements that can integrate different input signals and produce a range of output signals (7) (Figure 1). The way the IS ages and what are the changes that accompany and characterize this aging process have been the subject of intense studies in the last decades. In year 2000, our group proposed to call *inflammaging* the chronic, low-grade, sterile, inflammation that is almost universally present in old age and seems to be a hallmark of immunosenescence (6). The origins and sources of inflammaging are still matter of debate. In this review, we will discuss the possible involvement for the development and maintenance of inflammaging of a relatively newly described immunological phenomenon, i.e., innate immune memory or trained immunity. Trained immunity entails a cross-protection from different pathogens, and the first antigenic contact appears to be important in determining what kind of protection will be evoked. Therefore, it appears evident that type, intensity, and temporal sequence of antigens we are exposed to during the whole life are of extreme importance in determining the type of trained immunity that will rise up. More in general, the same concept is valid also for all the responses of the IS as a whole. The combination of these elements (type, intensity, and temporal sequence of antigens) is called “immunological biography” or *immunobiography*, and it can be considered unique for each individual. This uniqueness can explain how the same antigenic molecule, depending on the immunobiography of the host, can become either a strong or weak antigen or can induce tolerance. We will use the concept of immunobiography as a *fil rouge* of this review.

## Immunobiography and the Plasticity of the IS

**Figure 2:**
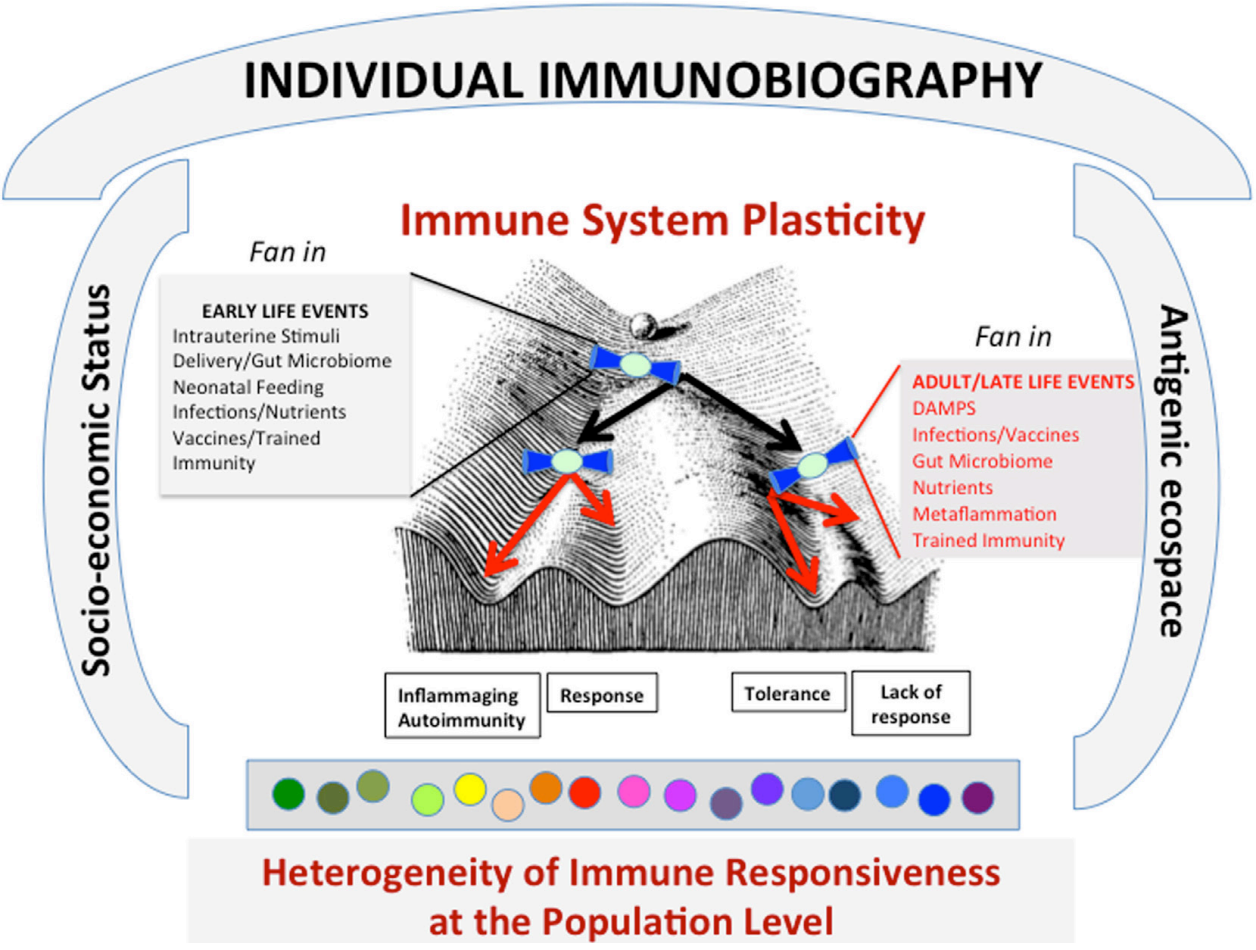
The lifelong personal history of antigenic exposure (Immunobiography), represented as a Waddington Landscape, modulates the immune response to specific antigens. The response to every single antigenic molecule depends on the conditions of immune system (IS) when it meets the antigen. A variety of conditions, including socioeconomical status and antigenic ecospace, impinge upon the IS. The antigens can be met during life under different environmental conditions that can shape the immune response (i.e., what slope the ball will follow in its path). It is surmised that these environmental conditions act and are integrated as “fan in” signal by a bow tie-like module of conserved elements. These conditions include both early life and adult-late life events. As a whole, this process can lead to the creation at population level of a large heterogeneity of immune responsiveness to specific antigens.

As mentioned above, a basic characteristic of the IS as a whole is *plasticity* (8), which means that the cells of the IS are not only able to recognize external and internal stressors but also to adapt and modify according to the variety of stimuli they are exposed to. To this regard, a large body of literature [reviewed in Ref. (8)] suggests that not only the type of molecular stimuli and their doses are critical but also their temporal sequence. The combination of these factors is integrated into a bow tie-shaped core (i.e., IS cells) to produce a variety of outputs (strong response, weak response, anergy, tolerance, memory, etc.). This integration occurs at every contact with an antigen/stressor. The whole history of antigenic encounters (and consequent integrations into *bow tie* architectural modules) or *immunobiography* can be represented as a Waddington Landscape (8) (Figure 2). Immunobiography starts *in utero* and continues lifelong since the very first day of life and is thus strongly influenced by early life events, as illustrated in Figure 2. In the event, the immune responses of each individual will be unique, owing to his or her immunological “history,” i.e., the summation (“immuneΣ”) and interaction of all the immunological experiences/stimuli. We argue that temporal and geographical dimensions, as well socioeconomic and psychological status, nutrition (oral tolerance and gut microbiota), and new potential source of unexpected epitopes produced by proteasome splicing (9) are integral component of immunobiography and could impinge upon the IS, thus inducing its continuous reshaping.

In conclusion, a variety of testable predictions derives from this conceptual framework, the most straightforward, suggesting that the immune responses to potentials antigens, including pathogens, food, and vaccines, will be quantitatively and qualitatively different according to the overall immune-biographical background of the host, including age, sex, lifestyle, socioeconomic and psychological status, and geography/genetics.

## Immunobiography and Inflammaging

We surmise that immunobiography is the best conceptual framework to understand the immune heterogeneity among individuals, including the difference in immune responses between men and women, and among different populations, whose genetics and IS have been molded by their evolutionary ecosystems and cultural habits (2). Moreover, the concept of immunobiography could explain the increased immune heterogeneity of old individuals and the age-related changes of the IS (i.e., immunosenescence and inflammaging). In fact, the IS undergoes a profound remodeling with age, contributing to the increased risk of infections, cancer, and autoimmune diseases (10). This remodeling affects both the innate and adaptive arms of the IS (11), and in general, it is thought to be a phenomenon associated with loss of functions and activities. However, this is not always true, as some features of the innate immunity seem to be preserved or even increased in immunosenescence (12, 13). In particular, inflammation is not dampened with age, and a low-grade, chronic, sterile inflammation (*inflammaging*) seems to be an almost universal phenomenon associated with advanced age (6, 14, 15).

The hyperproduction of innate immunity cytokines in elderly donors, including IL-6, TNF-α, and IL-1β, was first demonstrated in *in vitro* stimulated peripheral blood mononuclear cells (PBMCs) from aged people (16). The age-related activation of innate immunity was further confirmed in terms of blood levels of cytokines (17, 18) and chemokines (19–21). Accordingly, the age-related increase of pro- and anti-inflammatory mediators in peripheral blood was recently demonstrated on a large longitudinal cohort of Italians aged 20–102 years (22), underlying both the activation of innate immunity with age and the simultaneous activation of anti-inflammatory molecules, such as IL-10. Importantly, the presence of anti-inflammatory compensatory mechanisms was previously shown to be present also in centenarians (14), thus highlighting possible pathways of adaptation that likely favor longevity.

Since its very beginning, inflammaging was pigeonholed within an evolutionary framework where a central role of the macrophage was foreseen. This cell is indeed able to perform not only phagocytosis of foreign pathogens but also to produce a variety of soluble mediators, mainly but not exclusively pro-inflammatory (6, 23). The activation of this versatile cell (that is, now recognized to possess also a form of memory, see next paragraphs) likely accounts for the so-called physiological inflammation postulated since the beginning of the twentieth century by the great immunologist Il’ja Metchnikoff (24). Now, the available data indicate that other cell types (not necessarily belonging to the IS) can contribute to the setting up of the inflammaging, such as adipose and skeletal muscle cells. Moreover, an important contribution to inflammaging can arrive from senescent cells (1, 15), which are provided with a specific senescence-associated secretory phenotype characterized by the production of pro-inflammatory cytokines such as IL-6, IL-1β, IL-8 and chemokines such as CXCL1, CXCL2, matrix metalloproteinases, serine proteases, and regulators of plasminogen activators (PAI-1, PAI-2), etc. (25, 26). If the innate immune cells do not efficiently clear out these senescent cells, they can accumulate and contribute to the creation of a pro-inflammatory environment.

We have recently proposed that an age-related increase in the production of danger-associated molecular patterns (DAMPs) can impinge upon the level of inflammaging more importantly than pathogen-associated molecular patterns (PAMPs), through innate immune cells receptors, leading to innate inflammatory response (1). These DAMPs include, among others, high-mobility group B1 (HMGB1) protein, sodium monourate and uric acid crystals, oxidized fatty acids, and proteins. In particular, evidence exists for some specific molecules such as oxidized LDL, HMGB1, and uric acid. Oxidized LDL can train monocytes to secrete more pro-inflammatory cytokines (27, 28) (IL-6, IL-8, TNF, and MCP-1) and to express more pattern recognition receptors (PRRs) and LDL receptors (28). Mouse splenocytes that had been pretreated with HMGB1 responded with significantly higher TNF production when restimulated with PAMPs such as Pam3Cys, lipopolysaccharide (LPS), CpG, or other DAMPs like S100A12 (29). Finally, also uric acid appears to be able to prime the production of Il-1β and other pro-inflammatory cytokines in PBMC or monocytes (30).

The lifelong interaction between the gut microbiota and the IS could contribute to inflammaging. As recently summarized, host genetics, prenatal environment, and delivery mode can shape the newborn microbiome at birth (31). Moreover, a variety of other postnatal events such as antibiotic treatment, diet, exposure to infectious agents, among others can impinge upon and modify the development of the infant’s microbiome and IS, with long-term effects (risk for several diseases) in adult life. The age-related trajectory of the gut microbiota composition, from young adults to centenarians, and its possible contribution to inflammaging has been recently described (32, 33). A complex, lifelong remodeling of such a complex ecosystem emerged, where the decrease of potentially beneficial species and the increase of potential pathobionts related to systemic inflammation (32) is continuously counteracted by the increase of sub-dominant species, some of which likely exert a protective effects (33).

Inflammaging appears to be associated with decreasing health, but is also compatible with longevity, being present in centenarians. This apparent paradox can be understood in the light of immunobiography. According to this concept, a clinical history or an environmental circumstance could shape the IS to counteract inflammaging by setting up effective anti-inflammatory responses.

## Immunobiography, Trained Immunity, and the Memories of the IS

A paramount feature of the IS (and of immunobiography too) is *memory*, i.e., the capacity to give rise to a more rapid and efficient response at the second contact with a previously met antigen. Until few years ago, a tenet in immunology was that memory was an exclusive feature of the adaptive IS of vertebrates. A classic example reported since many years is the phenomenon of the “original antigenic sin” (34), which influences the type of response to a second challenge with a pathogen. Upon a primary response toward a pathogen (e.g., a virus), a subsequent exposure to the same pathogen elicits a secondary amplified and quicker response. However, if the second pathogen is very similar but not identical to the first, the IS can mistakenly identify the second pathogen as the first one encountered and progress to a classical memory response, which may be ineffective toward the second pathogen.

Actually, several observations have challenged this tenet, as examples of memory involving the innate branch of the IS were already reported since many decades (35). To this regard, it is known since long time that in plants and invertebrates, which only display innate immunity mechanisms, memory characteristics are present in the response to pathogens. In plants, a phenomenon called systemic acquired resistance (SAR) is well documented (36–38). This sort of primitive immunization protects plants for long periods of time against infections different from the one that elicited SAR, including viruses, bacteria, fungi, and oomycetes. In invertebrates, the existence of a form of memory where the information on a first encounter with a pathogen is stored and rapidly used on demand has now been demonstrated in a wide range of species (39, 40). Intriguingly, this type of responses can vary in degree and specificity in relation to different priming. Moreover, a phenomenon similar to allograft rejection after tissue transplantation has been demonstrated in some invertebrates (41, 42). For example, in second grafting experiments, leech responses to the second transplant were always faster and stronger than those occurring in first set grafting experiments. In second set experiments, two cell populations are evidenced, and some of them expressed CD56 and CD8-α and some others CD8-β and TNF-β allowing to postulate the existence of a sort of positive immune memory. In addition, the presence of CD8β- and TNF-β-positive cells in the graft area could suggest the existence of leukocyte-like cells that had previously responded to antigenic stimulation and have thus become able to respond rapidly to subsequent antigenic challenges. As a whole, these data support the idea that in invertebrates a sort of immunological memory exists even if with different features compared to the classical memory of the adaptive immunity present in vertebrates. In recent years, an ancestral network of cells with a thin, elongated morphology called “telocytes” (TCs) has been described in both invertebrates and vertebrates, including humans (see Box 1). As detailed in the Box 1, the TC ancestral network is able to integrate many different functions shared with players involved in trained immunity, such as complex innate immune responses, regenerative processes in wound healing, and secretion, so it is tempting to speculate that it might also play a role in trained immunity.

Box 1 **Telocytes (TCs) as possible players in trained immunity.**

Recently a new type of cellular system is described as ubiquitous in both vertebrates and invertebrates (40, 43–47). These cells named TCs are stromal cells strategically spread in various types of tissues from invertebrates up to humans. TCs are characterized by a very small spindle-shaped cell body, essentially occupied by a large nucleus, from which very long convoluted cytoplasmic processes, the telopods, originate. Thanks to these thread-like telopods, TCs communicate among themselves, with any other type of cells and interact with collagenic bundles, forming a key extensive intercellular network. The interaction among these different players take place directly by cell–cell contacts and indirectly *via* the release (in autocrine, paracrine, endocrine manner) of microvesicles and exosomes, which can transport a variety of soluble factors involved in the regulation of different physiological processes (47–51). TCs immunophenotype is quite complex. Apart the specific markers (co-expressed CD34/vimentin and Oct-4/c-kit) (44), these cells express markers of the immune-surveillance such as Toll-like receptors (TLRs) 4 and 5, allograft inflammatory factor-1 (Aif-1 also known as IBA-1) involved in inflammatory responses, adrenocorticotropic hormone implicated in the immune and neuroendocrine responses, and endogenous pro-inflammatory cytokines such as IL-18 (47). TCs respond to chemical or physical stimuli changing their morphology and behavior. These cells, by acquiring migratory phenotype, numerically increasing and overexpressing the previously mentioned factors, are able to rapidly move toward the injured area where they also participate in repair and regenerative processes (47, 52). Moreover, it has been observed in the leech *Hirudo medicinalis* that TCs originate from precursor circulating cells during the angiogenesis that ensues the graft rejection inflammatory phase. These invertebrate/vertebrate cells organized in a 3D network are equipped to function as an immune-neuroendocrine system. This evolutionarily conserved system is formed by resident cells working as outposts to signal the presence of non-self/damaged-self molecules and to alert the internal defenses of the organism. Owing to the fact that they are tissue resident, TC networks are able to respond promptly and faster than migrating immunocytes that need time to reach the stimulated (injected with LPS or injured) area.

Trained immunity appears to be based on innate immune cells that are also present in vertebrates. It was therefore conceivable that also in vertebrates similar phenomena were present. Consistently, studies performed in the past indicated the existence of an innate memory also in mice. In fact, vaccination with BCG was reported to protect mice against secondary infections with *Candida albicans* or *Schistosoma mansoni* through T cell-independent mechanisms (53), involving activated tissue macrophages (54). Moreover, infection with attenuated strains of *Candida* was observed to induce protection not only from reinfection with *Candida* itself but also from other pathogens such as *Staphylococcus aureus*, and this phenomenon was present also in athymic animals (55). More recently, it has been demonstrated that challenge of mice with CpG confers protection against *Listeria monocytogenes* infection (56). As a whole, it appears that in both invertebrates and vertebrates, innate immunity cells are provided with a capacity to respond more promptly to a second challenge, a feature that resemble the memory reactions typical of the adaptive immunity, with the crucial difference that such memory seems to be not limited to the specific antigen that triggered the first response. To describe this kind of innate memory, the group of Mihai Netea proposed the term “trained immunity” (57, 58). Trained immunity is evoked not only by microbial, viral, or fungal challenges (e.g., β-glucans, LPS) but also by molecules that are contained in vaccine adjuvants. Actually, adjuvants include TLR agonists such as monophosphoryl lipid A, CpG oligonucleotides, aluminum phosphate, or hydroxide salts. These adjuvants act mainly by inducing mild local inflammatory reactions that can boost the adaptive immune response toward the challenging antigen(s) (59). It has been shown that trained immunity is responsible for non-specific effects of vaccines such as BCG, OPV, and MMR (60, 61). It is known actually that these vaccines offer a protection from overall mortality that is not explained simply by the protection against the targeted pathogens (62). It is possible that this non-specific protection could be accounted for by the capability of adjuvants of inducing trained immunity responses (63).

At present, there is evidence that macrophages and NK cells are the main innate immune cells provided with this memory (64–66); however, also other cell types of both myeloid and lymphoid lineages (such as γ/δ T cells) seem to display similar features (67, 68), including NK-like CD8^+^ T cells, invariant NKT cells, and innate lymphoid cells (ILCs), even if more data are needed to clarify the underpinning mechanisms (see also next paragraph).

The basic molecular mechanisms involved in and responsible for the trained immunity memory appear to be of epigenetic nature. In fact, one of the mechanisms responsible of macrophages and dendritic cells (DCs) trained immunity is the capability to undergo epigenetic modifications following exposure to PAMPs or DAMPs (69). As it will be described in detail in Box 2, these epigenetic modifications induce high concentrations of inflammatory cytokines, including IL-1, IL-12, IL-18, and IL-23, which promote IL-17 and IFN-γ production by innate lymphocytes, including γδT cells, innate lymphoid cells (ILCs), and NKT cells, that exert protective effector functions against the second pathogen (58, 69, 70).

Box 2 **Trained immunity and epigenetics.**

There is evidence that trained immunity, at variance with adaptive immunity, does not imply genetic recombination, but relies upon epigenetic remodeling that influences gene expression profile without changing the DNA sequence of the cells. The first evidence that trained immunity is largely dependent from epigenetic mechanism derives from studies on plants (71).

Even though epigenetic changes tend to be maintained over time, they are less stable than the genetic rearrangement that occurs in adaptive immunity, and for this reason, trained immunity duration is shorter than adaptive immunity (that relies on clonal expansion of memory lymphocytes with specific receptors originated by genetic recombination). In general, the mechanism behind trained immunity can be recapitulated as follows: innate immunity cells, such as monocytes, macrophages, and NK cells, respond to antigenic stimuli by undergoing a shift in energy metabolism; this in turn causes an epigenetic rewriting that remains stable over time and have the potential to be inherited during cell differentiation. In particular, a shift of glucose metabolism from oxidative phosphorylation to aerobic glycolysis, increased glutamine metabolism, and cholesterol synthesis have been observed to play a crucial role in determining the establishment of the epigenetic modifications typical of the trained immunity phenomenon (72). Such epigenetic modifications lead to transcriptional programs that rewire the intracellular signaling of innate immune cells and induce an increase in the capacity to respond to the stimuli. A shift from phosphorylation to glycolysis has been observed in β-glucan-trained monocytes (70). There are different mechanisms by which a change in energy metabolism can impinge upon epigenetic setting. As an example, glycolysis results in higher ratios of NAD^+^/NADH, and this has been shown to activate Sirtuin 1 and 6 (73). Furthermore, it has been demonstrated that end products of glycolysis can inhibit histone deacetylases, thus causing genes to be more accessible (74).

Depending on the nature of the stimuli and the type of epigenetic modifications, cells maintain a hyperactivated phenotype for weeks or months. Accordingly, the specificity of the hyperactivation in response to the activating signal/agent is correlated with the epigenetic modification involved in the first response (58). Data obtained on monocytes indicate that upon vaccination with BCG, trained immunity was induced through the NOD2 receptor and mediated by increased histone 3 lysine 4 trimethylation (75). Epigenetic modifications can be triggered even in bone marrow precursors of immune cells. To this regard, a study on mice showed that bone marrow epigenetic remodeling of DC progenitors can be also stimulated by the gut microbiota (76). These data are of the utmost interest as they open a new perspective on the relationship between trained immunity, chronic inflammation, and a wide range of physiological and pathological conditions such as aging, obesity, and type 2 diabetes, where consistent changes in GM composition have been reported (32, 33, 77).

Beside histone modification, that is the prominent epigenetic mechanism involved in the trained immunity acquisition, other mechanisms are involved, such as DNA methylation and miRNA expression. DNA methylation was correlated with trained immunity after CMV infection (78, 79). In these studies, NK cells underwent large changes in the overall methylation profile, which altered profoundly their secretory capacity (78, 79). This result is particularly interesting when considering trained immunity under the perspective of aging, since it is known that the DNA methylation structure undergoes profound changes with age in all the tissues and organs (80). Accordingly, it will be of great interest to investigate the effect of such age-related modifications on trained immunity efficacy and plasticity.

A specific contribution is also played by microRNAs. They are short RNAs that play a critical role in influencing gene expression by silencing genes hierarchically high in the expression cascade of specific pathways. A critical characteristic of microRNAs is their long life in cells, thus providing a concrete contribution to the trained immunity setup (81). Among all the microRNAs, miR-155 is of particular interest, since its upregulation in response to external agents has been correlated with the activation of myeloid cells (82). Moreover, miR-155 constitutes a direct link between trained immunity and inflammaging since is one of the microRNAs involved in the regulation of inflammation (the so-called inflamma-mir) active in the aging process (83, 84).

The clear-cut distinction of innate and adaptive immunity based of the presence of memory is now much more blurred, and memory appears to be a shared property of the two branches of the IS, even if the memory of innate immunity (trained immunity) has different features. Therefore, the IS has at least two ways to remember previously encountered antigens. If and how these two “memories” do interact with each other is still unclear. They could act synergistically, or, on the contrary, trained immunity could dampen the adaptive one. This interaction could explain at least in part the heterogeneity of immune responses observed in the elderly. Urgent studies are needed to clarify this point. However, it is not known how trained immunity can change during aging and what contribution these possible changes can give to immunosenescence and inflammaging.

## Cells and Receptors Involved in Trained Immunity During Aging

In this paragraph, we will briefly discuss the present knowledge on the changes that occur with age in cells and their receptors presently known to be involved in trained immunity such as monocytes/macrophages, NK, and γδT cells.

Monocytes/macrophages are perhaps the most characterized cells involved in trained immunity, and it is well established that a great heterogeneity within this cell type does exist. Three different populations based on the differential expression of the LPS (CD14) and the FcIII (CD16) receptors (85) have been identified. This circulating monocyte pool dynamically changes during aging. In particular, CD14^+^ D16^+^ non-classical monocyte subset increases with age in healthy adults (21) but, importantly, displays reduced HLA-DR surface expression in elderly donors, suggesting a decline of antigen presentation function. Further, many data suggest that TLR expression and signaling efficiency in monocytes and DCs is modified during aging. A highly significant increase in TLR5-induced production of IL-8 from monocytes of older individuals has been reported along with an incomplete activation of NF-κB in response to TLR5 signaling (86). Moreover, in a large cohort of healthy human donors, peripheral blood monocytes from elderly donors showed a decreased expression and function of TLR1 (87). Similarly, reduced TLR levels and signaling responses in DCs were found (88). Interestingly, dysregulation of TLR3 in macrophages and lower production of IFN by DCs from elderly donors after infection with West Nile virus was reported (89). In addition, Metcalf et al. (90) have recently showed in a small cohort of donors that PBMCs from old subjects exhibited a slower immune response to TLR4, TLR7/8, and RIG-I agonists compared to cells from adult individuals. This was evident by the rapid induction of the IFN-signaling pathway in PBMCs from adults treated with different PRR agonists, including LPS among others. However, old subjects did produce higher levels of CCL1 in response to LPS and analogs.

Of note, TLR4, the receptor for LPS, is downregulated in macrophages that have been challenged with repeated exposures to low doses of LPS, a process known as endotoxin tolerance (91). Recently, it has been reported that the expression and activation of TLR4 induced by exposure to *Mycobacterium leprae* was downregulated upon the previous exposure to BCG (92). This suggests that trained immunity could involve TLR4 and that this involvement does not always entail activation, but also possible phenomena of tolerance. In particular, TLR4 and TLR2 can be responsible for tolerance, while other receptors like NOD2 and Dectin-1 can be responsible for trained immunity (30).

As far as NK cells, age-associated changes in phenotype and function have been described (93–95). First, NK cells express different functional TLRs (96, 97) recognizing bacterial PAMPs and activating their response (98–100). Other molecules, such as natural cytotoxicity receptors (NCRs), including NKp30 and NKp44, are key receptors in the recognition and the killing of virally infected or tumor cells. The recent identification of the cellular ligands for NKp44 and NKp30 such as exosomal proliferating cell nuclear antigen implicates that NCRs may also function as receptors for DAMPs (101). Therefore, the activation of NK cells could be amplified during aging due to the increased availability of DAMPs, according to the Garb-aging hypothesis (1). Further, immunosenescence is associated with the increase of CD56^dim^ NK cell subset, which expresses a mature phenotype, characterized by the augmented expression of markers such as CD57 (102) and KLRG1 (103, 104). The CD57 antigen (HNK-1, LEU-7) is also used to identify terminally differentiated “senescent” T cells with reduced proliferative capacity and altered functional properties as recently reviewed (105), but it seems to have a different expression pattern in NK cells. In fact, CD57 characterizes two typical NK subsets, i.e., the CD16^+^CD56^dim^ cytotoxic NK cells and the CD16^+^CD56^−^ inflammatory NK cells, whereas the CD16^−^CD56^bright^ regulatory NK cells do not express this marker even during chronic infections (102, 106). To this regard, infection with viruses including HIV and CMV could drive the expansion of CD57^+^NKG2C^high^ NK cells (107, 108). It has been proposed that CD57^+^NKG2C^high^ NK cells might represent human CMV-specific “memory” NK cells, thus highlighting the “adaptive characteristics” of NK cells (109). Remarkably, CD57^+^NKG2C^+^ NK cells expansion was observed in patients positive for both CMV and HIV, reaching levels >70% of all circulating NK cells, in comparison with patients who were positive only for either CMV or HIV (110). These data suggest that this NK subset may be trained by CMV and likely undergoes a great expansion when CMV reactivation occurs, a condition more frequently found in HIV-infected individuals. Hypothetically, a reactivation of latent virus can occur many times during aging and could stimulate CD57^+^NKG2C^high^ NK cells, therefore triggering expansion of this cell subset.

At variance, the subset of CD56^dim^ KLRG1^high^ NK cells is expanded in the elderly, displaying impaired cytotoxicity and proliferation as well as other features of senescence (103). Interestingly, KLRG1 or the killer cell lectin-like receptor G1 is also considered a marker for T cell senescence (111, 112) like CD57 molecule (113).

As a whole, these data suggest a convergence of adaptive and innate immunity during immunosenescence (114). A progression toward terminal differentiation (or senescence) of CD8^+^ T cells appears in fact to be associated with the acquisition of the hallmarks of innate-like T cells and the use of recently acquired NK cell receptors. These phenotypic, functional, and transcriptional changes would be a sort of compensation for functional deficits of conventional NK cells and T cells (115). Different health and environmental conditions, such as autoimmunity, inflammation, viral antigen re-exposure, or the presence of persistent tumor antigens, have been shown to allow the differentiation or “adaptation” of NK-like CD8^+^ T cells, as recently reviewed (116).

As far as γ/δ T cells, these cells can be activated independently from TCR and APCs. Receptors used by γ/δ T cells include NOTCH (117), NKG2D, and TLRs. To this regard, almost all TLRs were found in human γ/δ T cells (118). No data are currently available on age-related changes in expression or function of these receptors, even if it is known that a decline of total peripheral blood γ/δ T cell frequency occurs with age, along with changes in phenotype and TCR repertoire (95, 119), phenomena accentuated by CMV infection (120, 121). Interestingly, some data show that peripheral blood Vδ2(neg) γ/δ T cells are significantly increased in CMV-seropositive healthy individuals compared to CMV-seronegative controls in all age groups (122), thus reinforcing the idea that persistent antigenic load may modulate T cell repertoire with important effects also on innate immunity and inflammation (123).

Finally, it has been observed that also other innate immune cells such as group 2 innate lymphoid cells (ILC2s) display memory features (124). In the lung, ILC2s are stimulated by inhaled allergens and produce Th2-type cytokines inducing T cell-independent allergic lung inflammation. After the resolution of the inflammation, some ILC2s persist as allergen-experienced cells, can respond to unrelated allergens more potently than naive ILC2s, and exhibit a gene expression profile similar to that of memory T cells (124). Nothing is known at present on the possible modifications of the activity (and memory) of such cells during aging. Moreover, it is possible that also other innate immune cell types such as neutrophils or TCs (as proposed here) can be provided with memory features. Further studies are needed to test this hypothesis.

Overall, a complex scenario emerges for cells and receptors of innate immunity: some of them undergo consistent age-related impairment, while others are preserved or even hyper-regulated. Thus, trained immunity could dramatically change at advanced age due to the fact that some cell types (and their receptors) but not others undergo complex reshaping, possibly driven by the persistence of specific antigens (such as viral ones) or increased availability of DAMPs.

## Immunobiography Integrates Immunosenescence, Inflammaging, and Trained Immunity

The heterogeneity inherently present in any population is at the basis of a variety of important immunological, largely unclear aspects, such as the different responsiveness of individuals to various antigenic stimuli, i.e., bacteria, viruses, parasites, and vaccines. This heterogeneity also increases with age, thus becoming particularly important not only in immunology but also in gerontology and geriatrics, as it affects the risk of developing age-related diseases. The basic assumption and suggestion proposed here is that we have to pay particular attention to immunological anamnesis of each individual to reconstruct as accurately as possible the own immunobiography. Immunobiography goes beyond the simple, erratic measurement of immunological parameters (e.g., immunoglobulin level and lymphocyte subsets, or antibody titer in the blood at a certain time point). We think that an effort is required to put all the immunological information regarding a single person altogether, in a standardized and easily accessible way (chip?). This integrated perspective is at present largely neglected, likely because of a lack of standardized tools to collect the information necessary to describe the immunobiography of each individual. Information regarding the type of delivery (natural *vs* caesarian), of early nutrition (breast *vs* bottle feeding) and diet, the use of antibiotics, the composition of microbiota, the different types, sequence and number of infectious diseases and vaccinations, to mention only a few, is extremely informative in order to predict individual’s immune responses. Not less important are the data regarding ethnicity, socioeconomic, and psychological status that are an integral part of immunobiography.

As discussed all along this review, the knowledge on trained immunity in aging is still very scanty; accordingly, new experimental data are necessary to clarify the possible role of trained immunity in immunosenescence and inflammaging. At present, the available data, summarized in the previous paragraphs, are compatible with different possible scenarios. Trained immunity could undergo a functional impairment/decline with age, thus contributing to immunosenescence. However, it is also possible that trained immunity is hyperactivated with age, thus contributing to inflammaging and exerting deleterious effects on the onset of age-related chronic diseases (125, 126). Indeed, the main feature of cells of trained immunity is an enhanced production of pro-inflammatory cytokines, such as TNF-α, IFN-γ, and IL-1β in response to a subsequent challenge (127), and the receptors of innate immune cells can bind not only pathogen components but also “self” components (DAMPs) (1). LPS and other PAMPs can train monocytes/macrophages to become more pro-inflammatory when exposed to a second stimulus, but can also be rendered less responsive to pathogen or PAMPs through induction of tolerance or immunosuppression. The factors that determine whether a pathogen or a PAMP induces a state of trained immunity or tolerance/immunosuppression is unclear but may be influenced by the dose, timing, and nature of the exposure to the pathogen or PAMP. Moreover, factors related to immunological history and the life experiences could influence the trained immunity favoring one response or the other. This observation is particularly interesting for old persons characterized by a high heterogeneity that could be at least in part explained by different responses of monocytes/macrophages and other cells of the innate immunity to stimuli as a consequence of the different conditions they have experienced throughout life.

An urgent public health problem is to understand the immunological basis of the unresponsiveness to vaccines observed in a consistent percentage of elderly. On the basis of what we have discussed in this review, we surmise that the response to vaccines, in terms of both trained immunity and adaptive memory, is depending not only on the type/dose of immunological stimuli/vaccines that are encountered but also on the host immunobiography that shapes the responses of the IS. We surmise that this component, if adequately considered, will contribute to understand the poor responsiveness to vaccines and to newly encountered pathogens observed in a consistent number of elderly.

## Author Contributions

All authors checked literature data articles and reviews. MC, SS, PG, ME, and DM wrote the paper and critically discussed literature data; CF wrote the paper and coordinated the research.

## Conflict of Interest Statement

The authors declare that the research was conducted in the absence of any commercial or financial relationships that could be construed as a potential conflict of interest.
